# *cis*-Bis[*N*′-(4-bromo­benzo­yl)-*N*,*N*-dimethyl­thio­ureato-κ^2^ *O*,*S*]copper(II)

**DOI:** 10.1107/S1600536811015789

**Published:** 2011-04-29

**Authors:** Gün Binzet, Ulrich Flörke, Nevzat Külcü, Hakan Arslan

**Affiliations:** a Department of Chemistry, Faculty of Education, Mersin University, Mersin, TR 33169, Turkey; b Department of Chemistry, University of Paderborn, Paderborn D-33098, Germany; c Department of Chemistry, Faculty of Arts and Science, Mersin University, Mersin, TR 33343, Turkey; d Department of Chemistry, Emory University, Atlanta, GA 30322, USA

## Abstract

The asymmetric unit of the title compound, [Cu(C_10_H_10_BrN_2_OS)_2_], contains two independent complex mol­ecules with almost identical conformations. Two S and two O atoms form the coordination environment of the Cu atom, resulting in a slightly distorted square-planar coordination. The S atoms are in a *cis* configuration. The crystal structure is stabilized by weak inter­molecular C—H⋯Br hydrogen-bonding inter­actions.

## Related literature

For the synthesis of the title compound, see: Binzet *et al.* (2009 ▶); Emen *et al.* (2005 ▶). For complexes with thio­urea derivatives, see: Sacht *et al.* (2000 ▶); Arslan *et al.* (2009 ▶); Avşar *et al.* (2002 ▶, 2003 ▶); Mansuroğlu *et al.* (2008 ▶); Henderson *et al.* (2002 ▶). For related compounds, see: Arslan *et al.* (2003 ▶, 2006 ▶). For puckering parameters, see: Cremer & Pople (1975 ▶).
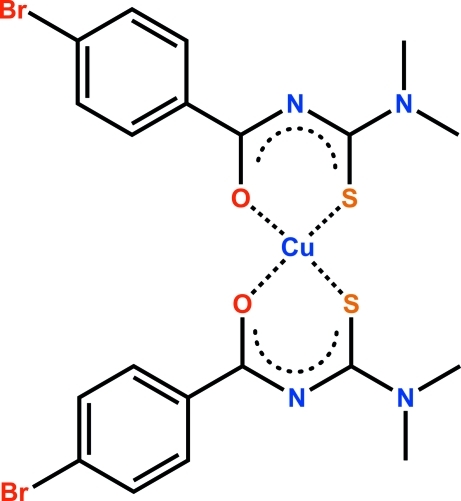


## Experimental

### 

#### Crystal data

[Cu(C_10_H_10_BrN_2_OS)_2_]
*M* *_r_* = 635.88
Triclinic, 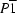

*a* = 9.1780 (11) Å
*b* = 11.0028 (13) Å
*c* = 23.241 (3) Å
α = 94.857 (2)°
β = 96.144 (3)°
γ = 95.095 (2)°
*V* = 2313.7 (5) Å^3^
*Z* = 4
Mo *K*α radiation
μ = 4.60 mm^−1^
*T* = 120 K
0.28 × 0.21 × 0.12 mm

#### Data collection

Bruker SMART APEX diffractometer
Absorption correction: multi-scan (*SADABS*; Sheldrick, 2004 ▶) *T*_min_ = 0.359, *T*_max_ = 0.608
20433 measured reflections
10926 independent reflections
8446 reflections with *I* > 2σ(*I*)
*R*_int_ = 0.029

#### Refinement

*R*[*F* ^2^ > 2σ(*F* ^2^)] = 0.042
*wR*(*F* ^2^) = 0.117
*S* = 1.00
10926 reflections
567 parameters
H-atom parameters constrained
Δρ_max_ = 0.98 e Å^−3^
Δρ_min_ = −0.84 e Å^−3^

### 

Data collection: *SMART* (Bruker, 2002 ▶); cell refinement: *SAINT* (Bruker, 2002 ▶); data reduction: *SAINT*; program(s) used to solve structure: *SHELXS97* (Sheldrick, 2008 ▶); program(s) used to refine structure: *SHELXL97* (Sheldrick, 2008 ▶); molecular graphics: *OLEX2* (Dolomanov *et al.*, 2009 ▶); software used to prepare material for publication: *SHELXTL* (Sheldrick, 2008 ▶), *OLEX2*, *publCIF* (Westrip, 2010 ▶) and *Mercury* (Macrae *et al.*, 2006 ▶).

## Supplementary Material

Crystal structure: contains datablocks I, global. DOI: 10.1107/S1600536811015789/bt5534sup1.cif

Structure factors: contains datablocks I. DOI: 10.1107/S1600536811015789/bt5534Isup2.hkl

Additional supplementary materials:  crystallographic information; 3D view; checkCIF report

## Figures and Tables

**Table 1:** Hydrogen-bond geometry (Å, °)

*D*—H⋯*A*	*D*—H	H⋯*A*	*D*⋯*A*	*D*—H⋯*A*
C13—H13*C*⋯Br2^i^	0.98	2.92	3.809 (4)	150
C19—H19*A*⋯Br4^ii^	0.95	2.91	3.858 (4)	174
C27—H27*A*⋯Br1^iii^	0.95	2.90	3.849 (4)	176
C37—H37*A*⋯Br3^iv^	0.95	2.93	3.845 (4)	163

Symmetry codes: (i) 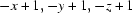; (ii) 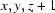; (iii) 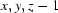; (iv) 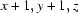.

**Fig. 1.**
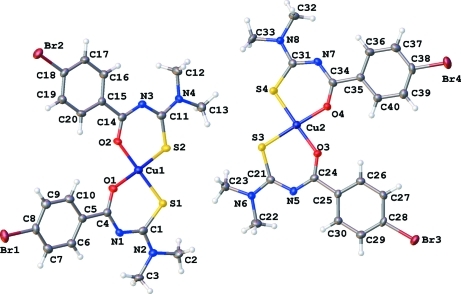
The molecular structure of the title compound, with displacement ellipsoids drawn at the 50% probability level.

**Fig. 2.**
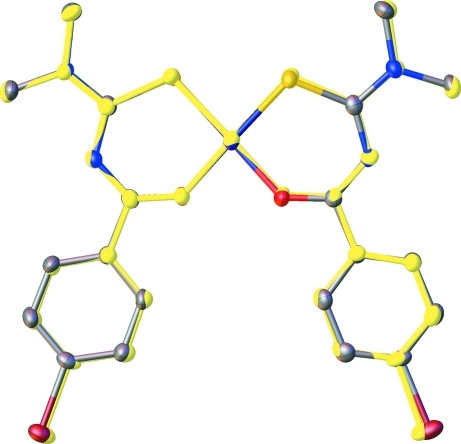
Overlay diagram of the two independent molecules in the crystal structure of the title compound.

## supplementary crystallographic information

### Comment

The ability of thiourea derivatives to complex with transition metal cations is well known (Arslan *et al.*, 2009; Avşar *et al.*, 2002, Arslan *et al.*, 2006). Thioureas are able to coordinate a range of metal centers as neutral ligands, monoanions or dianions (Sacht *et al.*, 2000; Henderson *et al.*, 2002). The oxygen, nitrogen and sulfur donors of thioureas provide a multitude of bonding possibilities.

In view of this information and in continuation of our research into the thiourea derivatives, we synthesized and characterized a series of substituted thiourea derivatives (Binzet *et al.*, 2009; Arslan *et al.*, 2003; Avşar *et al.*, 2003; Emen *et al.*, 2005; Mansuroğlu *et al.*, 2008). The title compound, *cis*-bis(*N*,*N*-dimethyl-*N*'-4-bromobenzoylthioureato)copper(II), (I), is another example of our synthesized thiourea derivatives that contains both aryl and alkyl groups.

There are two similar molecules in the asymmetric unit, so discussion will primarily focus on one of these independent molecules; see Fig. 1 and 2 for a view of one of the two independent molecules. There is very little difference between the bond lengths and angles of these molecules. The crystal structure of the title compound confirms *N*,*N*-dimethyl-*N*'-4-bromobenzoylthiourea ligand is a bidentate chelating ligand, coordinating to the copper atom through the thiocarbonyl and carbonyl groups. The central copper atom shows slightly distorted square-planar coordination and the sulfur atoms are in a *cis* configuration. The maximum deviations from the S_2_O_2_ mean plane are 0.065 (3) Å for oxygen, 0.054 (1) Å for sulfur and 0.000 (1) Å for copper. The Cu1/S2/C11/N3/C14/O2 ring adopts an envelope conformation with puckering parameter Q = 0.2593 (19) Å, θ = 121.0 (7)°, φ = 181.3 (8) ° (Cremer & Pople, 1975). Cu—O [average 1.857 (3) Å] and Cu—S [average 2.1442 (10) Å] bond lengths are in the expected ranges. The dihedral angle between these chelate planes of 6.23 (10)° indicates slight distortion from square planar towards tetrahedral geometry. C–O, C–S and C–N bond lengths of the complex suggest considerable electronic delocalization in the chelate rings. The bond lengths of the carbonyl O1–C4 1.265 (4) Å; O2–C14 1.270 (4) Å and thiocarbonyl S1–C1 1.738 (4) Å; S2–C11 1.734 (4) Å groups lie between those for double and single bonds, similar to related structures (Arslan *et al.* 2003, 2006; Avşar *et al.*, 2003; Binzet *et al.*, 2009), while both C–O and C–S bond lengths are typical of double bonds in the free ligand. The other bond lengths in title compound show normal values.

### Experimental

A solution of 4-bromobenzoyl chloride (0.005 M) in acetone (50 cm^3^) was added dropwise to a suspension of potassium thiocyanate (0.005 M) in anhydrous acetone (50 cm^3^). The reaction mixture was heated under reflux for 30 min and then cooled to room temperature. A solution of dimethylamine (0.005 M) in acetone (30 cm^3^) was added and the resulting mixture was stirred for 2 h. Hydrochloric acid (0.1 N, 300 cm^3^) was added and the solution filtered. The solid product was washed with water and purified by recrystallization from ethanol: dichloromethane (1: 2). M.p.: 222–224 °C. C_20_H_20_Br_2_CuN_4_O_2_S_2_: C, 37.78; H, 3.17; N, 8.81. Found: C, 37.97; H, 3.20; N, 8.79%.

### Refinement

H atom positions were clearly derived from difference Fourier maps and refined using a riding model, fixing the bond lengths at 0.98 and 0.95 Å for CH_3_ and CH(aromatic), respectively. The displacement parameters of the H atoms were constrained with *U*_iso_(H) = 1.2*U*_eq_ (C) or 1.5*U*_eq_ (methyl C).

### Crystal data

**Table:**

[Cu(C_10_H_10_BrN_2_OS)_2_]	*Z* = 4
*M**_r_* = 635.88	*F*(000) = 1260
Triclinic, *P*1	*D*_x_ = 1.825 Mg m^−^^3^
Hall symbol: -P 1	Mo *K*α radiation, λ = 0.71073 Å
*a* = 9.1780 (11) Å	Cell parameters from 3977 reflections
*b* = 11.0028 (13) Å	θ = 2.3–28.7°
*c* = 23.241 (3) Å	µ = 4.60 mm^−^^1^
α = 94.857 (2)°	*T* = 120 K
β = 96.144 (3)°	Prism, red
γ = 95.095 (2)°	0.28 × 0.21 × 0.12 mm
*V* = 2313.7 (5) Å^3^	

### Data collection

**Table:**

Bruker SMART APEX diffractometer	10926 independent reflections
Radiation source: sealed tube	8446 reflections with *I* > 2σ(*I*)
graphite	*R*_int_ = 0.029
φ and ω scans	θ_max_ = 27.9°, θ_min_ = 0.9°
Absorption correction: multi-scan (*SADABS*; Sheldrick, 2004)	*h* = −11→12
*T*_min_ = 0.359, *T*_max_ = 0.608	*k* = −14→14
20433 measured reflections	*l* = −30→28

### Refinement

**Table:**

Refinement on *F*^2^	Primary atom site location: structure-invariant direct methods
Least-squares matrix: full	Secondary atom site location: difference Fourier map
*R*[*F*^2^ > 2σ(*F*^2^)] = 0.042	Hydrogen site location: difference Fourier map
*wR*(*F*^2^) = 0.117	H-atom parameters constrained
*S* = 1.00	*w* = 1/[σ^2^(*F*_o_^2^) + (0.0696*P*)^2^] where *P* = (*F*_o_^2^ + 2*F*_c_^2^)/3
10926 reflections	(Δ/σ)_max_ = 0.001
567 parameters	Δρ_max_ = 0.98 e Å^−^^3^
0 restraints	Δρ_min_ = −0.84 e Å^−^^3^

### Special details

**Table:**

Geometry. All e.s.d.'s (except the e.s.d. in the dihedral angle between two l.s. planes) are estimated using the full covariance matrix. The cell e.s.d.'s are taken into account individually in the estimation of e.s.d.'s in distances, angles and torsion angles; correlations between e.s.d.'s in cell parameters are only used when they are defined by crystal symmetry. An approximate (isotropic) treatment of cell e.s.d.'s is used for estimating e.s.d.'s involving l.s. planes.
Refinement. Refinement of *F*^2^ against ALL reflections. The weighted *R*-factor *wR* and goodness of fit *S* are based on *F*^2^, conventional *R*-factors *R* are based on *F*, with *F* set to zero for negative *F*^2^. The threshold expression of *F*^2^ > σ(*F*^2^) is used only for calculating *R*-factors(gt) *etc*. and is not relevant to the choice of reflections for refinement. *R*-factors based on *F*^2^ are statistically about twice as large as those based on *F*, and *R*- factors based on ALL data will be even larger.

### Fractional atomic coordinates and isotropic or equivalent isotropic displacement parameters (Å^2^)

**Table:**

	*x*	*y*	*z*	*U*_iso_*/*U*_eq_	
Cu1	0.28252 (5)	0.12213 (4)	0.428728 (19)	0.02274 (11)	
Br1	0.37548 (5)	−0.19864 (4)	0.753935 (17)	0.03539 (11)	
Br2	1.00293 (4)	0.48331 (4)	0.664265 (18)	0.03169 (10)	
S1	0.13289 (10)	−0.01269 (8)	0.37520 (4)	0.02310 (19)	
S2	0.26287 (10)	0.23785 (8)	0.35908 (4)	0.02323 (19)	
O1	0.2935 (3)	0.0289 (2)	0.49212 (11)	0.0255 (6)	
O2	0.4210 (3)	0.2305 (2)	0.47515 (11)	0.0250 (5)	
N1	0.0876 (3)	−0.1172 (3)	0.47730 (13)	0.0210 (6)	
N2	−0.0790 (3)	−0.1706 (3)	0.39781 (13)	0.0240 (6)	
N3	0.5181 (3)	0.3709 (3)	0.41560 (13)	0.0214 (6)	
N4	0.4318 (3)	0.4184 (3)	0.32595 (13)	0.0240 (7)	
C1	0.0445 (4)	−0.1031 (3)	0.42136 (16)	0.0214 (7)	
C2	−0.1409 (4)	−0.1633 (4)	0.33765 (17)	0.0308 (9)	
H2A	−0.1534	−0.0776	0.3314	0.046*	
H2B	−0.2368	−0.2121	0.3300	0.046*	
H2C	−0.0741	−0.1952	0.3112	0.046*	
C3	−0.1613 (4)	−0.2547 (4)	0.43166 (18)	0.0294 (8)	
H3A	−0.1034	−0.2607	0.4691	0.044*	
H3B	−0.1799	−0.3361	0.4101	0.044*	
H3C	−0.2553	−0.2234	0.4383	0.044*	
C4	0.2079 (4)	−0.0569 (3)	0.50652 (15)	0.0200 (7)	
C5	0.2476 (4)	−0.0921 (3)	0.56630 (15)	0.0211 (7)	
C6	0.1521 (4)	−0.1724 (3)	0.59169 (16)	0.0241 (7)	
H6A	0.0620	−0.2067	0.5701	0.029*	
C7	0.1876 (4)	−0.2026 (3)	0.64818 (17)	0.0278 (8)	
H7A	0.1217	−0.2559	0.6656	0.033*	
C8	0.3206 (4)	−0.1534 (3)	0.67833 (15)	0.0252 (8)	
C9	0.4182 (4)	−0.0729 (3)	0.65460 (17)	0.0259 (8)	
H9A	0.5086	−0.0393	0.6762	0.031*	
C10	0.3797 (4)	−0.0429 (3)	0.59820 (16)	0.0247 (8)	
H10A	0.4447	0.0120	0.5813	0.030*	
C11	0.4137 (4)	0.3472 (3)	0.36911 (16)	0.0213 (7)	
C12	0.5545 (4)	0.5137 (4)	0.33028 (18)	0.0339 (9)	
H12A	0.5697	0.5556	0.3697	0.051*	
H12B	0.5324	0.5730	0.3022	0.051*	
H12C	0.6440	0.4765	0.3219	0.051*	
C13	0.3332 (4)	0.4071 (4)	0.27193 (16)	0.0289 (8)	
H13A	0.2945	0.3211	0.2620	0.043*	
H13B	0.3873	0.4355	0.2407	0.043*	
H13C	0.2513	0.4571	0.2768	0.043*	
C14	0.5131 (4)	0.3156 (3)	0.46358 (15)	0.0207 (7)	
C15	0.6319 (4)	0.3585 (3)	0.51186 (15)	0.0198 (7)	
C16	0.7371 (4)	0.4549 (3)	0.50670 (16)	0.0269 (8)	
H16A	0.7326	0.4953	0.4721	0.032*	
C17	0.8489 (4)	0.4928 (4)	0.55177 (17)	0.0291 (8)	
H17A	0.9212	0.5581	0.5481	0.035*	
C18	0.8525 (4)	0.4340 (3)	0.60154 (16)	0.0234 (7)	
C19	0.7489 (4)	0.3378 (3)	0.60788 (17)	0.0272 (8)	
H19A	0.7529	0.2980	0.6427	0.033*	
C20	0.6396 (4)	0.3010 (3)	0.56235 (16)	0.0254 (8)	
H20A	0.5684	0.2348	0.5660	0.031*	
Cu2	0.35911 (5)	0.18310 (4)	0.070914 (19)	0.02242 (11)	
Br3	0.00952 (4)	−0.42199 (4)	−0.175362 (17)	0.03082 (10)	
Br4	0.73008 (5)	0.17128 (4)	−0.253135 (18)	0.03850 (12)	
S3	0.25683 (10)	0.14812 (8)	0.14730 (4)	0.02390 (19)	
S4	0.45871 (11)	0.35186 (8)	0.11731 (4)	0.0259 (2)	
O3	0.2718 (3)	0.0415 (2)	0.02839 (11)	0.0256 (6)	
O4	0.4483 (3)	0.2068 (2)	0.00413 (11)	0.0256 (6)	
N5	0.0961 (3)	−0.0513 (3)	0.08088 (13)	0.0216 (6)	
N6	0.0270 (3)	0.0084 (3)	0.16917 (13)	0.0252 (7)	
N7	0.6098 (3)	0.3874 (3)	0.02166 (13)	0.0228 (6)	
N8	0.6605 (3)	0.5243 (3)	0.10071 (13)	0.0235 (6)	
C21	0.1191 (4)	0.0278 (3)	0.12893 (15)	0.0205 (7)	
C22	−0.0890 (5)	−0.0925 (4)	0.15936 (19)	0.0373 (10)	
H22A	−0.1277	−0.1036	0.1181	0.056*	
H22B	−0.1685	−0.0742	0.1827	0.056*	
H22C	−0.0490	−0.1678	0.1706	0.056*	
C23	0.0390 (4)	0.0848 (4)	0.22442 (16)	0.0285 (8)	
H23A	0.1327	0.0755	0.2473	0.043*	
H23B	−0.0426	0.0592	0.2461	0.043*	
H23C	0.0349	0.1708	0.2169	0.043*	
C24	0.1727 (4)	−0.0415 (3)	0.03654 (15)	0.0200 (7)	
C25	0.1355 (4)	−0.1392 (3)	−0.01296 (15)	0.0196 (7)	
C26	0.2139 (4)	−0.1347 (3)	−0.06115 (16)	0.0228 (7)	
H26A	0.2928	−0.0726	−0.0612	0.027*	
C27	0.1772 (4)	−0.2202 (3)	−0.10896 (16)	0.0234 (7)	
H27A	0.2297	−0.2169	−0.1419	0.028*	
C28	0.0628 (4)	−0.3107 (3)	−0.10794 (15)	0.0222 (7)	
C29	−0.0135 (4)	−0.3181 (3)	−0.06067 (17)	0.0278 (8)	
H29A	−0.0906	−0.3817	−0.0605	0.033*	
C30	0.0227 (4)	−0.2317 (3)	−0.01284 (16)	0.0244 (8)	
H30A	−0.0301	−0.2361	0.0200	0.029*	
C31	0.5819 (4)	0.4225 (3)	0.07554 (15)	0.0205 (7)	
C32	0.7652 (4)	0.5938 (3)	0.06970 (18)	0.0315 (9)	
H32A	0.8643	0.5695	0.0797	0.047*	
H32B	0.7645	0.6816	0.0809	0.047*	
H32C	0.7371	0.5768	0.0277	0.047*	
C33	0.6478 (5)	0.5730 (4)	0.15988 (17)	0.0311 (9)	
H33A	0.5494	0.6001	0.1620	0.047*	
H33B	0.7223	0.6428	0.1713	0.047*	
H33C	0.6631	0.5091	0.1862	0.047*	
C34	0.5439 (4)	0.2865 (3)	−0.00926 (15)	0.0211 (7)	
C35	0.5885 (4)	0.2613 (3)	−0.06848 (15)	0.0211 (7)	
C36	0.6950 (4)	0.3395 (3)	−0.08950 (17)	0.0281 (8)	
H36A	0.7396	0.4107	−0.0660	0.034*	
C37	0.7353 (4)	0.3128 (4)	−0.14478 (17)	0.0309 (9)	
H37A	0.8079	0.3654	−0.1592	0.037*	
C38	0.6697 (4)	0.2099 (4)	−0.17848 (15)	0.0266 (8)	
C39	0.5635 (4)	0.1312 (3)	−0.15925 (17)	0.0276 (8)	
H39A	0.5190	0.0605	−0.1831	0.033*	
C40	0.5238 (4)	0.1586 (3)	−0.10393 (16)	0.0244 (8)	
H40A	0.4507	0.1057	−0.0900	0.029*	

### Atomic displacement parameters (Å^2^)

**Table:**

	*U*^11^	*U*^22^	*U*^33^	*U*^12^	*U*^13^	*U*^23^
Cu1	0.0229 (2)	0.0228 (2)	0.0215 (2)	−0.00394 (17)	0.00288 (18)	0.00245 (17)
Br1	0.0406 (2)	0.0475 (3)	0.0205 (2)	0.01112 (19)	0.00464 (17)	0.00856 (17)
Br2	0.0305 (2)	0.0327 (2)	0.0279 (2)	−0.00079 (16)	−0.00602 (16)	−0.00411 (16)
S1	0.0268 (5)	0.0224 (4)	0.0186 (4)	−0.0055 (3)	0.0029 (3)	0.0018 (3)
S2	0.0219 (4)	0.0224 (4)	0.0236 (5)	−0.0054 (3)	−0.0014 (3)	0.0046 (3)
O1	0.0259 (13)	0.0274 (13)	0.0217 (14)	−0.0076 (11)	0.0007 (11)	0.0068 (11)
O2	0.0240 (13)	0.0286 (13)	0.0205 (13)	−0.0107 (11)	0.0024 (10)	0.0048 (11)
N1	0.0221 (15)	0.0215 (14)	0.0199 (15)	−0.0009 (12)	0.0054 (12)	0.0037 (12)
N2	0.0229 (15)	0.0240 (15)	0.0233 (16)	−0.0053 (12)	0.0017 (12)	0.0012 (12)
N3	0.0220 (15)	0.0208 (14)	0.0201 (16)	−0.0034 (12)	0.0020 (12)	0.0009 (12)
N4	0.0270 (16)	0.0261 (16)	0.0177 (16)	−0.0058 (13)	0.0018 (13)	0.0051 (12)
C1	0.0214 (17)	0.0166 (16)	0.0260 (19)	0.0018 (13)	0.0051 (14)	−0.0014 (14)
C2	0.026 (2)	0.035 (2)	0.027 (2)	−0.0080 (17)	−0.0052 (16)	0.0016 (17)
C3	0.028 (2)	0.0276 (19)	0.030 (2)	−0.0130 (16)	0.0052 (16)	0.0012 (16)
C4	0.0207 (17)	0.0198 (16)	0.0193 (18)	0.0015 (13)	0.0033 (14)	0.0000 (13)
C5	0.0243 (18)	0.0193 (16)	0.0195 (18)	0.0010 (14)	0.0031 (14)	0.0012 (13)
C6	0.0234 (18)	0.0239 (18)	0.0242 (19)	−0.0016 (14)	0.0030 (15)	0.0024 (15)
C7	0.030 (2)	0.0271 (19)	0.028 (2)	0.0007 (16)	0.0085 (16)	0.0053 (16)
C8	0.031 (2)	0.0302 (19)	0.0159 (18)	0.0079 (16)	0.0032 (15)	0.0030 (15)
C9	0.0213 (18)	0.0299 (19)	0.027 (2)	0.0029 (15)	0.0028 (15)	0.0030 (16)
C10	0.0208 (18)	0.0254 (18)	0.028 (2)	−0.0015 (14)	0.0062 (15)	0.0027 (15)
C11	0.0196 (17)	0.0184 (16)	0.0259 (19)	0.0016 (13)	0.0040 (14)	0.0011 (14)
C12	0.034 (2)	0.037 (2)	0.028 (2)	−0.0113 (18)	0.0008 (17)	0.0107 (18)
C13	0.031 (2)	0.033 (2)	0.022 (2)	−0.0026 (16)	−0.0005 (16)	0.0062 (16)
C14	0.0206 (17)	0.0194 (16)	0.0217 (18)	0.0014 (13)	0.0045 (14)	−0.0024 (14)
C15	0.0216 (17)	0.0199 (16)	0.0181 (17)	0.0014 (13)	0.0046 (14)	0.0000 (13)
C16	0.034 (2)	0.0258 (19)	0.0196 (19)	−0.0066 (16)	−0.0014 (15)	0.0066 (15)
C17	0.033 (2)	0.0273 (19)	0.024 (2)	−0.0115 (16)	0.0042 (16)	0.0006 (15)
C18	0.0233 (18)	0.0228 (17)	0.0214 (19)	0.0000 (14)	−0.0018 (14)	−0.0051 (14)
C19	0.033 (2)	0.0256 (19)	0.0214 (19)	−0.0040 (16)	0.0014 (16)	0.0049 (15)
C20	0.029 (2)	0.0203 (17)	0.026 (2)	−0.0085 (14)	0.0051 (15)	0.0047 (15)
Cu2	0.0251 (2)	0.0211 (2)	0.0201 (2)	−0.00261 (17)	0.00350 (18)	0.00064 (17)
Br3	0.0370 (2)	0.0287 (2)	0.0227 (2)	−0.00348 (16)	−0.00316 (16)	−0.00510 (15)
Br4	0.0525 (3)	0.0426 (2)	0.0223 (2)	0.0032 (2)	0.01448 (19)	0.00322 (17)
S3	0.0280 (5)	0.0236 (4)	0.0182 (4)	−0.0060 (4)	0.0041 (4)	−0.0015 (3)
S4	0.0328 (5)	0.0213 (4)	0.0224 (5)	−0.0072 (4)	0.0093 (4)	−0.0027 (4)
O3	0.0311 (14)	0.0226 (13)	0.0208 (14)	−0.0098 (11)	0.0064 (11)	−0.0025 (10)
O4	0.0329 (14)	0.0243 (13)	0.0183 (13)	−0.0085 (11)	0.0073 (11)	0.0006 (10)
N5	0.0221 (15)	0.0241 (15)	0.0170 (15)	−0.0012 (12)	−0.0009 (12)	0.0007 (12)
N6	0.0260 (16)	0.0285 (16)	0.0195 (16)	−0.0073 (13)	0.0062 (13)	−0.0013 (13)
N7	0.0256 (16)	0.0223 (15)	0.0210 (16)	−0.0020 (12)	0.0067 (12)	0.0040 (12)
N8	0.0295 (16)	0.0210 (15)	0.0188 (16)	−0.0060 (12)	0.0054 (13)	−0.0003 (12)
C21	0.0209 (17)	0.0198 (16)	0.0207 (18)	−0.0002 (13)	0.0011 (14)	0.0051 (14)
C22	0.033 (2)	0.042 (2)	0.034 (2)	−0.0151 (19)	0.0097 (18)	−0.0004 (19)
C23	0.032 (2)	0.0298 (19)	0.024 (2)	−0.0039 (16)	0.0099 (16)	−0.0006 (16)
C24	0.0202 (17)	0.0179 (16)	0.0204 (18)	−0.0020 (13)	−0.0005 (14)	0.0018 (13)
C25	0.0227 (18)	0.0207 (16)	0.0143 (17)	−0.0001 (13)	−0.0005 (13)	0.0007 (13)
C26	0.0221 (18)	0.0221 (17)	0.0233 (19)	−0.0040 (14)	0.0026 (14)	0.0033 (14)
C27	0.0265 (19)	0.0267 (18)	0.0175 (18)	−0.0018 (15)	0.0069 (14)	0.0042 (14)
C28	0.0280 (19)	0.0214 (17)	0.0146 (17)	0.0016 (14)	−0.0051 (14)	−0.0029 (13)
C29	0.027 (2)	0.0268 (19)	0.027 (2)	−0.0080 (15)	0.0014 (16)	0.0015 (16)
C30	0.0275 (19)	0.0243 (18)	0.0206 (19)	−0.0044 (15)	0.0048 (15)	0.0019 (14)
C31	0.0213 (17)	0.0211 (17)	0.0188 (18)	0.0004 (13)	0.0007 (14)	0.0038 (14)
C32	0.037 (2)	0.0241 (19)	0.032 (2)	−0.0093 (17)	0.0070 (18)	0.0040 (16)
C33	0.040 (2)	0.0255 (19)	0.024 (2)	−0.0085 (17)	0.0031 (17)	−0.0088 (16)
C34	0.0250 (18)	0.0190 (16)	0.0199 (18)	0.0008 (14)	0.0046 (14)	0.0052 (14)
C35	0.0252 (18)	0.0188 (16)	0.0193 (18)	0.0000 (14)	0.0039 (14)	0.0030 (13)
C36	0.032 (2)	0.0240 (18)	0.027 (2)	−0.0070 (16)	0.0071 (16)	0.0016 (15)
C37	0.034 (2)	0.029 (2)	0.030 (2)	−0.0044 (17)	0.0117 (17)	0.0039 (16)
C38	0.035 (2)	0.032 (2)	0.0140 (18)	0.0074 (17)	0.0064 (15)	0.0038 (15)
C39	0.035 (2)	0.0251 (18)	0.022 (2)	−0.0010 (16)	0.0028 (16)	0.0014 (15)
C40	0.0286 (19)	0.0218 (17)	0.0222 (19)	−0.0025 (15)	0.0047 (15)	0.0014 (14)

### Geometric parameters (Å, °)

**Table:**

Cu1—O2	1.850 (3)	Cu2—O3	1.842 (2)
Cu1—O1	1.864 (3)	Cu2—O4	1.857 (2)
Cu1—S1	2.1433 (10)	Cu2—S3	2.1423 (10)
Cu1—S2	2.1451 (10)	Cu2—S4	2.1435 (10)
Br1—C8	1.895 (4)	Br3—C28	1.897 (3)
Br2—C18	1.904 (4)	Br4—C38	1.903 (4)
S1—C1	1.738 (4)	S3—C21	1.739 (4)
S2—C11	1.734 (4)	S4—C31	1.741 (4)
O1—C4	1.265 (4)	O3—C24	1.271 (4)
O2—C14	1.270 (4)	O4—C34	1.266 (4)
N1—C4	1.324 (4)	N5—C24	1.315 (4)
N1—C1	1.344 (5)	N5—C21	1.342 (4)
N2—C1	1.336 (4)	N6—C21	1.344 (4)
N2—C2	1.464 (5)	N6—C22	1.454 (5)
N2—C3	1.474 (5)	N6—C23	1.462 (5)
N3—C14	1.318 (5)	N7—C34	1.329 (5)
N3—C11	1.356 (4)	N7—C31	1.337 (4)
N4—C11	1.339 (4)	N8—C31	1.332 (4)
N4—C13	1.456 (5)	N8—C33	1.453 (5)
N4—C12	1.459 (5)	N8—C32	1.463 (5)
C2—H2A	0.9800	C22—H22A	0.9800
C2—H2B	0.9800	C22—H22B	0.9800
C2—H2C	0.9800	C22—H22C	0.9800
C3—H3A	0.9800	C23—H23A	0.9800
C3—H3B	0.9800	C23—H23B	0.9800
C3—H3C	0.9800	C23—H23C	0.9800
C4—C5	1.490 (5)	C24—C25	1.496 (5)
C5—C10	1.391 (5)	C25—C30	1.386 (5)
C5—C6	1.400 (5)	C25—C26	1.397 (5)
C6—C7	1.393 (5)	C26—C27	1.386 (5)
C6—H6A	0.9500	C26—H26A	0.9500
C7—C8	1.382 (5)	C27—C28	1.385 (5)
C7—H7A	0.9500	C27—H27A	0.9500
C8—C9	1.393 (5)	C28—C29	1.369 (5)
C9—C10	1.395 (5)	C29—C30	1.391 (5)
C9—H9A	0.9500	C29—H29A	0.9500
C10—H10A	0.9500	C30—H30A	0.9500
C12—H12A	0.9800	C32—H32A	0.9800
C12—H12B	0.9800	C32—H32B	0.9800
C12—H12C	0.9800	C32—H32C	0.9800
C13—H13A	0.9800	C33—H33A	0.9800
C13—H13B	0.9800	C33—H33B	0.9800
C13—H13C	0.9800	C33—H33C	0.9800
C14—C15	1.493 (5)	C34—C35	1.488 (5)
C15—C20	1.377 (5)	C35—C40	1.389 (5)
C15—C16	1.391 (5)	C35—C36	1.399 (5)
C16—C17	1.394 (5)	C36—C37	1.390 (5)
C16—H16A	0.9500	C36—H36A	0.9500
C17—C18	1.371 (5)	C37—C38	1.373 (5)
C17—H17A	0.9500	C37—H37A	0.9500
C18—C19	1.387 (5)	C38—C39	1.381 (5)
C19—C20	1.385 (5)	C39—C40	1.390 (5)
C19—H19A	0.9500	C39—H39A	0.9500
C20—H20A	0.9500	C40—H40A	0.9500
			
O2—Cu1—O1	84.47 (11)	O3—Cu2—O4	83.73 (11)
O2—Cu1—S1	176.03 (9)	O3—Cu2—S3	94.44 (8)
O1—Cu1—S1	93.66 (8)	O4—Cu2—S3	177.74 (8)
O2—Cu1—S2	94.24 (8)	O3—Cu2—S4	177.64 (9)
O1—Cu1—S2	176.26 (9)	O4—Cu2—S4	94.60 (8)
S1—Cu1—S2	87.84 (4)	S3—Cu2—S4	87.27 (4)
C1—S1—Cu1	107.27 (13)	C21—S3—Cu2	108.25 (12)
C11—S2—Cu1	108.03 (13)	C31—S4—Cu2	109.18 (12)
C4—O1—Cu1	132.3 (2)	C24—O3—Cu2	134.5 (2)
C14—O2—Cu1	132.4 (2)	C34—O4—Cu2	134.3 (2)
C4—N1—C1	122.7 (3)	C24—N5—C21	123.0 (3)
C1—N2—C2	121.9 (3)	C21—N6—C22	120.4 (3)
C1—N2—C3	121.5 (3)	C21—N6—C23	122.5 (3)
C2—N2—C3	116.6 (3)	C22—N6—C23	117.1 (3)
C14—N3—C11	123.1 (3)	C34—N7—C31	123.3 (3)
C11—N4—C13	123.4 (3)	C31—N8—C33	122.6 (3)
C11—N4—C12	121.3 (3)	C31—N8—C32	121.2 (3)
C13—N4—C12	115.3 (3)	C33—N8—C32	116.2 (3)
N2—C1—N1	115.7 (3)	N5—C21—N6	115.7 (3)
N2—C1—S1	116.1 (3)	N5—C21—S3	128.6 (3)
N1—C1—S1	128.2 (3)	N6—C21—S3	115.7 (3)
N2—C2—H2A	109.5	N6—C22—H22A	109.5
N2—C2—H2B	109.5	N6—C22—H22B	109.5
H2A—C2—H2B	109.5	H22A—C22—H22B	109.5
N2—C2—H2C	109.5	N6—C22—H22C	109.5
H2A—C2—H2C	109.5	H22A—C22—H22C	109.5
H2B—C2—H2C	109.5	H22B—C22—H22C	109.5
N2—C3—H3A	109.5	N6—C23—H23A	109.5
N2—C3—H3B	109.5	N6—C23—H23B	109.5
H3A—C3—H3B	109.5	H23A—C23—H23B	109.5
N2—C3—H3C	109.5	N6—C23—H23C	109.5
H3A—C3—H3C	109.5	H23A—C23—H23C	109.5
H3B—C3—H3C	109.5	H23B—C23—H23C	109.5
O1—C4—N1	129.9 (3)	O3—C24—N5	129.5 (3)
O1—C4—C5	114.2 (3)	O3—C24—C25	114.3 (3)
N1—C4—C5	115.8 (3)	N5—C24—C25	116.1 (3)
C10—C5—C6	119.0 (3)	C30—C25—C26	119.4 (3)
C10—C5—C4	120.1 (3)	C30—C25—C24	121.7 (3)
C6—C5—C4	120.9 (3)	C26—C25—C24	118.9 (3)
C7—C6—C5	120.9 (4)	C27—C26—C25	120.3 (3)
C7—C6—H6A	119.6	C27—C26—H26A	119.8
C5—C6—H6A	119.6	C25—C26—H26A	119.8
C8—C7—C6	118.6 (4)	C28—C27—C26	119.0 (3)
C8—C7—H7A	120.7	C28—C27—H27A	120.5
C6—C7—H7A	120.7	C26—C27—H27A	120.5
C7—C8—C9	122.2 (3)	C29—C28—C27	121.5 (3)
C7—C8—Br1	119.5 (3)	C29—C28—Br3	120.4 (3)
C9—C8—Br1	118.3 (3)	C27—C28—Br3	118.0 (3)
C8—C9—C10	118.2 (4)	C28—C29—C30	119.4 (3)
C8—C9—H9A	120.9	C28—C29—H29A	120.3
C10—C9—H9A	120.9	C30—C29—H29A	120.3
C5—C10—C9	121.1 (3)	C25—C30—C29	120.3 (3)
C5—C10—H10A	119.4	C25—C30—H30A	119.9
C9—C10—H10A	119.4	C29—C30—H30A	119.9
N4—C11—N3	114.6 (3)	N8—C31—N7	115.4 (3)
N4—C11—S2	117.0 (3)	N8—C31—S4	115.9 (3)
N3—C11—S2	128.4 (3)	N7—C31—S4	128.7 (3)
N4—C12—H12A	109.5	N8—C32—H32A	109.5
N4—C12—H12B	109.5	N8—C32—H32B	109.5
H12A—C12—H12B	109.5	H32A—C32—H32B	109.5
N4—C12—H12C	109.5	N8—C32—H32C	109.5
H12A—C12—H12C	109.5	H32A—C32—H32C	109.5
H12B—C12—H12C	109.5	H32B—C32—H32C	109.5
N4—C13—H13A	109.5	N8—C33—H33A	109.5
N4—C13—H13B	109.5	N8—C33—H33B	109.5
H13A—C13—H13B	109.5	H33A—C33—H33B	109.5
N4—C13—H13C	109.5	N8—C33—H33C	109.5
H13A—C13—H13C	109.5	H33A—C33—H33C	109.5
H13B—C13—H13C	109.5	H33B—C33—H33C	109.5
O2—C14—N3	129.5 (3)	O4—C34—N7	129.7 (3)
O2—C14—C15	114.5 (3)	O4—C34—C35	114.2 (3)
N3—C14—C15	116.0 (3)	N7—C34—C35	116.1 (3)
C20—C15—C16	119.0 (3)	C40—C35—C36	118.9 (3)
C20—C15—C14	119.8 (3)	C40—C35—C34	119.9 (3)
C16—C15—C14	121.3 (3)	C36—C35—C34	121.3 (3)
C15—C16—C17	120.7 (3)	C37—C36—C35	119.9 (4)
C15—C16—H16A	119.7	C37—C36—H36A	120.1
C17—C16—H16A	119.7	C35—C36—H36A	120.1
C18—C17—C16	118.8 (3)	C38—C37—C36	119.6 (4)
C18—C17—H17A	120.6	C38—C37—H37A	120.2
C16—C17—H17A	120.6	C36—C37—H37A	120.2
C17—C18—C19	121.7 (3)	C37—C38—C39	122.0 (3)
C17—C18—Br2	120.1 (3)	C37—C38—Br4	119.3 (3)
C19—C18—Br2	118.1 (3)	C39—C38—Br4	118.7 (3)
C20—C19—C18	118.5 (3)	C38—C39—C40	118.0 (4)
C20—C19—H19A	120.8	C38—C39—H39A	121.0
C18—C19—H19A	120.8	C40—C39—H39A	121.0
C15—C20—C19	121.4 (3)	C35—C40—C39	121.6 (3)
C15—C20—H20A	119.3	C35—C40—H40A	119.2
C19—C20—H20A	119.3	C39—C40—H40A	119.2
			
O1—Cu1—S1—C1	22.70 (14)	O3—Cu2—S3—C21	−11.88 (15)
S2—Cu1—S1—C1	−153.87 (12)	S4—Cu2—S3—C21	166.49 (13)
O2—Cu1—S2—C11	16.75 (15)	O4—Cu2—S4—C31	−4.72 (15)
S1—Cu1—S2—C11	−159.88 (13)	S3—Cu2—S4—C31	174.00 (13)
O2—Cu1—O1—C4	161.0 (3)	O4—Cu2—O3—C24	−173.4 (3)
S1—Cu1—O1—C4	−22.5 (3)	S3—Cu2—O3—C24	7.9 (3)
O1—Cu1—O2—C14	159.3 (3)	O3—Cu2—O4—C34	−176.0 (4)
S2—Cu1—O2—C14	−24.2 (3)	S4—Cu2—O4—C34	5.6 (3)
C2—N2—C1—N1	178.8 (3)	C24—N5—C21—N6	177.0 (3)
C3—N2—C1—N1	0.0 (5)	C24—N5—C21—S3	−5.8 (5)
C2—N2—C1—S1	−3.6 (4)	C22—N6—C21—N5	−0.2 (5)
C3—N2—C1—S1	177.6 (3)	C23—N6—C21—N5	178.8 (3)
C4—N1—C1—N2	179.7 (3)	C22—N6—C21—S3	−177.8 (3)
C4—N1—C1—S1	2.5 (5)	C23—N6—C21—S3	1.2 (5)
Cu1—S1—C1—N2	162.9 (2)	Cu2—S3—C21—N5	14.2 (4)
Cu1—S1—C1—N1	−19.8 (3)	Cu2—S3—C21—N6	−168.6 (2)
Cu1—O1—C4—N1	7.8 (6)	Cu2—O3—C24—N5	0.8 (6)
Cu1—O1—C4—C5	−170.2 (2)	Cu2—O3—C24—C25	178.3 (2)
C1—N1—C4—O1	7.7 (6)	C21—N5—C24—O3	−4.0 (6)
C1—N1—C4—C5	−174.3 (3)	C21—N5—C24—C25	178.5 (3)
O1—C4—C5—C10	−8.1 (5)	O3—C24—C25—C30	−175.4 (3)
N1—C4—C5—C10	173.6 (3)	N5—C24—C25—C30	2.5 (5)
O1—C4—C5—C6	170.5 (3)	O3—C24—C25—C26	2.7 (5)
N1—C4—C5—C6	−7.9 (5)	N5—C24—C25—C26	−179.4 (3)
C10—C5—C6—C7	0.3 (5)	C30—C25—C26—C27	1.4 (5)
C4—C5—C6—C7	−178.3 (3)	C24—C25—C26—C27	−176.7 (3)
C5—C6—C7—C8	−1.2 (5)	C25—C26—C27—C28	−0.6 (5)
C6—C7—C8—C9	1.4 (5)	C26—C27—C28—C29	−0.6 (6)
C6—C7—C8—Br1	−177.2 (3)	C26—C27—C28—Br3	177.2 (3)
C7—C8—C9—C10	−0.8 (5)	C27—C28—C29—C30	1.1 (6)
Br1—C8—C9—C10	177.9 (3)	Br3—C28—C29—C30	−176.8 (3)
C6—C5—C10—C9	0.4 (5)	C26—C25—C30—C29	−1.0 (5)
C4—C5—C10—C9	179.0 (3)	C24—C25—C30—C29	177.1 (3)
C8—C9—C10—C5	−0.2 (5)	C28—C29—C30—C25	−0.3 (6)
C13—N4—C11—N3	179.3 (3)	C33—N8—C31—N7	−177.8 (3)
C12—N4—C11—N3	−0.4 (5)	C32—N8—C31—N7	2.2 (5)
C13—N4—C11—S2	−0.8 (5)	C33—N8—C31—S4	1.4 (5)
C12—N4—C11—S2	179.6 (3)	C32—N8—C31—S4	−178.5 (3)
C14—N3—C11—N4	175.5 (3)	C34—N7—C31—N8	178.5 (3)
C14—N3—C11—S2	−4.5 (5)	C34—N7—C31—S4	−0.6 (5)
Cu1—S2—C11—N4	171.5 (2)	Cu2—S4—C31—N8	−175.2 (2)
Cu1—S2—C11—N3	−8.5 (3)	Cu2—S4—C31—N7	3.8 (4)
Cu1—O2—C14—N3	16.2 (6)	Cu2—O4—C34—N7	−3.4 (6)
Cu1—O2—C14—C15	−164.1 (2)	Cu2—O4—C34—C35	176.1 (2)
C11—N3—C14—O2	3.5 (6)	C31—N7—C34—O4	−0.5 (6)
C11—N3—C14—C15	−176.3 (3)	C31—N7—C34—C35	−179.9 (3)
O2—C14—C15—C20	3.8 (5)	O4—C34—C35—C40	1.1 (5)
N3—C14—C15—C20	−176.5 (3)	N7—C34—C35—C40	−179.3 (3)
O2—C14—C15—C16	−177.1 (3)	O4—C34—C35—C36	−178.9 (3)
N3—C14—C15—C16	2.7 (5)	N7—C34—C35—C36	0.7 (5)
C20—C15—C16—C17	0.2 (6)	C40—C35—C36—C37	−0.7 (6)
C14—C15—C16—C17	−179.0 (3)	C34—C35—C36—C37	179.3 (3)
C15—C16—C17—C18	−0.6 (6)	C35—C36—C37—C38	0.3 (6)
C16—C17—C18—C19	0.5 (6)	C36—C37—C38—C39	0.1 (6)
C16—C17—C18—Br2	−179.8 (3)	C36—C37—C38—Br4	−178.0 (3)
C17—C18—C19—C20	0.0 (6)	C37—C38—C39—C40	−0.1 (6)
Br2—C18—C19—C20	−179.7 (3)	Br4—C38—C39—C40	178.0 (3)
C16—C15—C20—C19	0.4 (5)	C36—C35—C40—C39	0.7 (6)
C14—C15—C20—C19	179.6 (3)	C34—C35—C40—C39	−179.3 (3)
C18—C19—C20—C15	−0.5 (6)	C38—C39—C40—C35	−0.3 (6)

### Hydrogen-bond geometry (Å, °)

**Table:**

*D*—H···*A*	*D*—H	H···*A*	*D*···*A*	*D*—H···*A*
C13—H13C···Br2^i^	0.98	2.92	3.809 (4)	150
C19—H19A···Br4^ii^	0.95	2.91	3.858 (4)	174
C27—H27A···Br1^iii^	0.95	2.90	3.849 (4)	176
C37—H37A···Br3^iv^	0.95	2.93	3.845 (4)	163

Symmetry codes: (i) −*x*+1, −*y*+1, −*z*+1; (ii) *x*, *y*, *z*+1; (iii) *x*, *y*, *z*−1; (iv) *x*+1, *y*+1, *z*.

## References

[bb1] Arslan, H., Duran, N., Börekçi, G., Özer, C. K. & Akbay, C. (2009). *Molecules*, **14**, 519–527. 10.3390/molecules14010519 PMC6253946 19169199

[bb2] Arslan, H., Flörke, U., Külcü, N. & Emen, F. M. (2006). *J. Coord. Chem.* **59**, 223–228.

[bb3] Arslan, H., Vanderveer, D., Emen, F. M. & Külcü, N. (2003). *Z. Kristallogr. New Cryst. Struct.* **218**, 479–480.

[bb4] Avşar, G., Arslan, H., Haupt, H.-J. & Külcü, N. (2003). *Turk. J. Chem.* **27**, 281–285.

[bb5] Avşar, G., Külcü, N. & Arslan, H. (2002). *Turk. J. Chem.* **26**, 607–615.

[bb6] Binzet, G., Külcü, N., Flörke, U. & Arslan, H. (2009). *J. Coord. Chem.* **62**, 3454–3462.

[bb7] Bruker (2002). *SMART*, *SAINT* and *SADABS* Bruker AXS Inc., Madison, Wisconsin, USA.

[bb8] Cremer, D. & Pople, J. A. (1975). *J. Am. Chem. Soc.* **97**, 1354–1358.

[bb9] Dolomanov, O. V., Bourhis, L. J., Gildea, R. J., Howard, J. A. K. & Puschmann, H. (2009). *J. Appl. Cryst.* **42**, 339–341.

[bb10] Emen, F. M., Arslan, H., Külcü, N., Flörke, U. & Duran, N. (2005). *Pol. J. Chem.* **79**, 1615–1626.

[bb11] Henderson, W., Nicholson, K., Dinger, M. B. & Bennett, R. L. (2002). *Inorg. Chim. Acta*, **338**, 210–218.

[bb12] Macrae, C. F., Edgington, P. R., McCabe, P., Pidcock, E., Shields, G. P., Taylor, R., Towler, M. & van de Streek, J. (2006). *J. Appl. Cryst.* **39**, 453–457.

[bb13] Mansuroğlu, D. S., Arslan, H., Flörke, U. & Külcü, N. (2008). *J. Coord. Chem.* **61**, 3134–3146.

[bb14] Sacht, C., Datt, M. S., Otto, S. & Roodt, A. (2000). *J. Chem. Soc. Dalton Trans.* pp. 4579–4585.

[bb15] Sheldrick, G. M. (2004). *SADABS* University of Göttingen, Germany.

[bb16] Sheldrick, G. M. (2008). *Acta Cryst.* A**64**, 112–122. 10.1107/S0108767307043930 18156677

[bb17] Westrip, S. P. (2010). *J. Appl. Cryst.* **43**, 920–925.

